# A Retrospective Literature Review of Eating Disorder Research (1990–2021): Application of Bibliometrics and Topical Trends

**DOI:** 10.3390/ijerph19137710

**Published:** 2022-06-23

**Authors:** Eunhye Park, Woo-Hyuk Kim

**Affiliations:** 1 Department of Food Nutrition, College of BioNano Technology, Gachon University, Seongnam 13120, Korea; epark@gachon.ac.kr; 2 Department of Consumer Science, College of Commerce and Public Affairs, Incheon National University, Incheon 22012, Korea

**Keywords:** bibliometrics, eating disorder, big data, topic modeling, literature review

## Abstract

Despite the growing importance of eating disorders in society and academic literature, only a few bibliometric review studies using bibliometric analysis were available. Hence, this study aimed to explore and uncover hidden research topics and patterns in articles in terms of eating disorders over the last 30 years. In total, 4111 articles on eating disorders were analyzed using bibliometrics, network analyses, and structural topic modeling as the basis of mixed methods. In addition to general statistics about the journal, several key research topics, such as eating disorder (ED) treatment, ED symptoms, factors triggering ED, family related factors, eating behaviors, and social factors, were found based on topic correlations. This study found the key research variables that are frequently studied with EDs, such as AN, BN, BED, and ARFID. This study may help clinicians comprehend important risk factors associated with EDs. Moreover, the findings about key ED research topics and their association can be helpful for future studies to construct a comprehensive ED research framework. To our knowledge, this is the first study to use topic modeling in an academic journal on EDs and examine the diversity in ED research over 30 years of published research.

## 1. Introduction

According to the National Eating Disorder Association (NEDA) [1], about 30 million people in the United States (US) suffer from eating disorders (EDs), including anorexia nervosa (AN), bulimia nervosa (BN), or binge eating disorder (BED) at least once in their lifetime. NEDA also reported that people who have AN at some point in their lives account for nearly 1% of females and 0.3% of males, while those who have BN account for roughly 1.5% of females and 0.1% of males [1]. BED is more common than AN and BN, with roughly 3.5 percent of women and 2.0 percent of men experiencing BED in their lives [1].

EDs are complicated illnesses that induce a variety of mental and physical health symptoms, significantly increasing the disease burden [2]. Through a national survey with the representative US adults, Udo and Grilo [3] uncovered that psychiatric disorders, especially substance use disorders, mood disorders, and anxiety disorders, were more prevalent among groups of the US adults suffering from three types of EDs (i.e., AN, BN, and BED) than those without specific EDs. Moreover, they discovered EDs could increase rates of somatic comorbidities, such as arthritis, hypertension, sleep problems, and high cholesterol [3].

ED research has developed into a diverse and specialized field owing to the complicated nature of these diseases, having made practical and theoretical contributions in various areas, such as the conceptualization of EDs [4,5], diagnosis [6,7], treatment and intervention [8,9], and risk factors associated with EDs [10,11]. For continuous academic development, researchers must actively communicate and collaborate with other scholars, even in other disciplines or subject areas, if necessary [12]. However, the rapid growth of specialized and multidisciplinary ED research may challenge researchers, especially young researchers, to understand the progress in the sub-research topics of ED research [13]. An overall understanding of ED research can be even more difficult as trends and research foci in ED studies may change over time in line with the evolving concepts and environments around ED [14].

Formal or casual in-person meetings or researchers’ individual efforts to search for information online or offline can be helpful for scholarly communication. With the advent of online databases and bibliometrics, the development of academic achievement can be easily structured, and information exchange among researchers can be traced [15]. Therefore, bibliometric methods can provide practical, impartial approaches to evaluating the publication profiles of a journal and research outcomes [13,16]. Citation analysis, a part of bibliometrics, can demonstrate how scholars communicate to conduct research and revolutionize ED research [12]. This study also implemented topic modeling to discover prominent research themes and associations among research topics. A retrospective literature review of ED research can provide a broad understanding of key research areas and trends over time. Based on the findings of this study, researchers and practitioners can comprehend areas of research that have hitherto been influential or areas of study that will require greater input from fellow researchers and practitioners in the future.

Previous studies conducted an extensive review of ED research published in 1980, 1990, and 2000 through collaboration between statistical and field experts [17,18]. Based on solid empirical evidence, the authors successfully illustrate the historical changes in methodological approaches and hypothesis testing and draw useful implications for academic stakeholders, such as researchers, reviewers, and editorial boards. However, no studies have been conducted since 2006 that evaluate the bibliographic data and research output of ED studies to diagnose academic progress and establish sustainable development. Hence, the current study aims to summarize the history of articles on eating disorders by showcasing its intellectual structure according to authors, citations, and, more importantly, research perspectives on the topic since 1990. Of the numerous journals that accept ED-related research, this study focused on the ED-specialized journal, *International Journal of Eating Disorders* (*IJED*), which has been one of the most influential journals in the field of ED over the past three decades. The research questions (RQs) were as follows:

RQ 1. What are the general characteristics of ED studies published in articles on eating disorders?
RQ 2. How was ED research developed? Specifically, we suggest the following specific research questions in relation to RQ2:
  RQ 2-1. Which articles on eating disorders received the most attention from other researchers?
  RQ 2-2. What was the status of the researchers’ collaboration in developing ED research?
  RQ 2-3. Which papers have been widely cited as grounds for ED research?
RQ 3. What topics are being actively studied in the field of ED and how has the popularity of these topics changed over time?

To our knowledge, our study is the first to apply bibliometrics and topic modeling to content in an academic journal addressing EDs to explore the diversity of studies on the subject over 30 years. Hence, this study introduced bibliometric methods to the field of ED research. The methodology and findings of this study are expected to contribute to the continuous development of ED research and inspire researchers in the field.

## 2. Methodology

### 2.1. Data Collection

The Web of Science (WoS) database was used to collect all articles published in *International Journal of Eating Disorders* (*IJED)* between January 1990 and August 2021. For data collection, this study chose one representative ED-related journal, *IJED.* According to Shah et al. [19], *Archives of General Psychiatry*, *American Journal of Psychiatry*, *International Journal of Eating Disorders*, and *Psychological Medicine* published the most influential, in other words, most cited, ED research. Out of these journals, *IJED* was the only ED-specialized journal. Although there are other prestigious ED-specialized journals with high impact factors, such as *Eating disorders, Journal of eating disorders, European Eating Disorders Review*, this study focused on *IJED.* Since the main foci of the aforementioned ED-specialized journals can vary, we chose one journal to control the influence of journal features on bibliometric results. Papers published in *Eating Disorders*, for example, have been available in the WoS since 2012, and papers published in *Journal of Eating Disorders* have been available since 2017. Because the availability of papers published in various ED journals varies, the topic summary results may be influenced accordingly.

In the WoS database, all article-related information, such as keywords, abstracts, volumes, issues, and page counts; information about the authors, including names, affiliations, and ORCID; and citation information, such as the number of citations and cited references, were retrieved. Of the 4160 articles retrieved from the WoS, 49 that did not contain essential article information (i.e., year of publication, volume, or issue) were excluded, leaving 4111 articles for data analysis. By following the common practices of previous reviews and bibliometric studies [17,20,21], this study divided the dataset into three periods to discover the key characteristics of the journal in each decade: 1990–1999, 2000–2009, and 2010–August 2021.

### 2.2. Bibliometric Analysis

This study applied two computer-assisted tools to efficiently capture the massive amount of journal-related information over the past 30 years: (1) the R-based bibliometric package “bibliometrix”, and (2) structural topic modeling (STM), an R-based text mining tool.

Traditionally, bibliographic data have been analyzed manually, which largely relies on the researchers’ subjective judgments of the data and requires a significant amount of time for data analysis. However, as the size of the data increases and the reproducibility of the results becomes more important, automatic analysis techniques such as bibliometrics have been widely applied [15]. Bibliometrics are statistical or quantitative analyses of a comprehensive range of the data in the literature and have been widely applied in various academic disciplines [21,22,23,24]. Bibliometric analysis tools often provide statistical summaries of journals or articles, author characteristics, institution or country characteristics, and citation characteristics. This study conducted bibliometrics using the R studio (R version 3.6.3 (1 September 2021) with the R-package, “bibliometrix”. (version 3.1.4) [25]. The general statistics of the journals and citation characteristics were examined using this package.

The “bibliometrix package” was used for network analysis to identify collaborative author relationships and co-citation patterns. For the author collaborative relationship network, *each node of the network indicates the author of the* articles on eating disorders, *and the researchers who collaborated are connected with a line.* Only key edges and 30 nodes consisting of key authors were used for network visualization to improve the visibility of the network. Each node represents the cited reference for the co-citation network, and the top 30 giant nodes are included for network visualization. For both networks, betweenness centrality was calculated because of its good performance in detecting influential nodes in the network [26,27]. The sizes of the nodes and labels are proportional to their degree in the network. For both the author’s collaborative relationship network and the co-citation network, community detection was performed using the default setting to identify the key groups.

### 2.3. Topic Modeling

To identify major research topics in articles published in *Eating Disorders*, we conducted *topic modeling*, which is computer-based text analysis. Because the key information about each article is concentrated in the title, abstract, and keywords, these three pieces of information were combined and analyzed for text mining. Python3 (version 3.7.3) was used for data cleaning to improve the quality of the text mining results. We performed text cleaning using two Python packages: Natural Language Toolkit, better known as “NLTK (version 3.4.4)”, and Gensim (version 3.8.0).

For topic modeling, an STM algorithm was applied with the “stm” package (version 1.3.6) in R [28]. Topic modeling is a machine learning approach that automates the modeling process with multiple iterations. However, for machines to produce results, users of the topic modeling algorithm must determine the optimal number of topics for the dataset and provide that information as input. If the number of topics is too small, machine-generated topics may not capture important sub-research topics or research trends. If the number of topics is too large, on the other hand, multiple similar topics can be generated redundantly. To identify the proper range of topics, held-out likelihood scores were calculated for different topics and used as a quantitative index. To ensure the quality of topic modeling results, the authors of this study performed an additional review of the machine-generated results. That is, the two authors of this paper (both have expertise in the implemented methods, and one is a registered dietitian) have manually reviewed the top words and abstracts highly associated with each topic to confirm whether the results were reasonable and interpretable. Following these procedures, a topic model was built with 47 topics.

Each topic consisted of a series of terms that addressed specific themes. The algorithm examined the associations between the terms in the dataset and terms often used in the same document or context were grouped together. Because topic modeling is probabilistic modeling, the machine calculates each term’s probability of being associated with the 47 topics and each document’s probability of the same to obtain a probability score called the *topic weight (β)*. Because the sum of 47 topic weights per document is always one, a topic closely related to one document has a topic weight close to one, whereas the topic weight given low relatedness is close to zero.

Because the machine computes the probability of one document being associated with all 47 topics, the associations among the topics could be examined as well. More specifically, topics that often occurred together in the same document had strong associations, and topic networks were created based on these associations. To do so, the “topicCorr” function in the “stm” package was applied. This process provided us with insights into more general and broader trends in topics in the selected article sample. Based on topic correlation, the modularity optimization method (“cluster_optimal” in the igraph package in R) was used to apply a community detection algorithm with a high modularity score. The modularity score was applied to discover the optimal community structure in complex networks.

STM has a function of testing the effects of document-level metadata on topic weights, which is available as an “estimateEffect” function in the “stm” package [29]. To simulate the effects of document covariates on topic weights, a component of document-level metadata is included as a parameter (*X*) instead of a global mean prior applicable for all documents [29]. The topic weight was referred to from a multivariate normal linear distribution [29]. With the “estimateEffect” function, we compared topic weights across the three periods to uncover shifts in the popularity of topics over time. Specifically, the “pointestimate” method was used to estimate the expected topic weights (*β)* by each value of covariates (that is, three-time points by each decade), and the “difference” method was used to calculate the difference in the expected topic weights and confidence intervals. Since this approach can contrast two groups with binary data, topic weights of the 1990s were compared with those of the 2000s and 2010s, and the 2000s with the 2010s.

### 2.4. Research Topic Classification

Two metrics, overall popularity and historical trends, were utilized for topic classification. Topic estimates were used as indicators to determine the overall popularity of topics. The topics ranking within the top 25% by median topic estimates were classified into “high”, between the 25th percentile and the 75th percentile into “moderate”, and below 25% into “low”.

**Figure 1:**
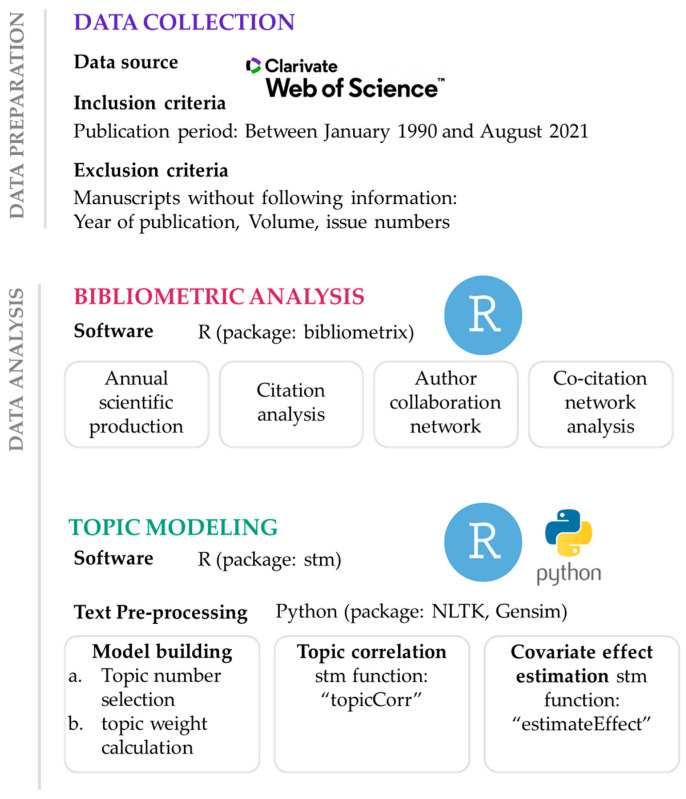
Summary of methodology.

For historical trend classification, topic weight estimates were compared every decade based on 95% confidence intervals as the basis for determining one of the following historical trend classifications: “increasing”, “decreasing”, and “constant”. Historical trends of topics were classified as “increasing” (or “decreasing”) if the low and high confidence intervals did not contain zeros and their weights increased (or decreased) over time. If the low and high confidence intervals contain zeros, those topics are classified as “constant”. Figure 1 illustrates a summary of the implemented methods.

## 3. Result

### 3.1. General Characteristics of Articles in Terms of Eating Disorders

**Figure 2:**
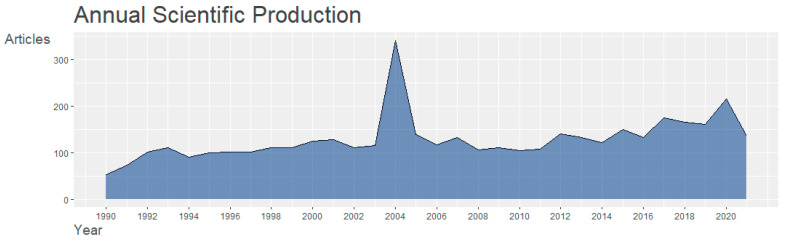
The number of articles published in *Eating Disorders*.

As illustrated in Figure 2, an average of 132 articles were published in the *ED’s journals* each year between 1990 and 2020 (2020 was excluded from the average calculation as this year was not complete at the time of data analysis). The number of publications peaked in 2004 at 341, nearly three times the annual average. Although the change in the number of articles over time was not significant, it tended to increase at the end of each 10 years. Of the 4111 articles, the number of articles published between 1990 and 1999 was 952, 1419 between 2000 and 2009, and 1740 between 2010 and 2021. Of these years, 2004 emerged as the peak year for the number of publications.

### 3.2. Citation Characteristics

Table 1 lists the top 20 articles of each decade that received the most citations regarding the characteristics of citations. The article that received the most citations throughout the 30 years, was “Assessment of Eating Disorders: Interview or Self-Report Questionnaire?” [30]. Among the articles published in the first decade, those on scale development tended to be cited frequently. In the second decade, the article “The Effect of Experimental Presentation of Thin Media Images on Body Satisfaction: A Meta-Analytic Review” [31] received the most citations, and studies involving systematic reviews or meta-analyses were cited most frequently. Among articles published since 2010, “Psychometric Evaluation of the Eating Disorder Examination and Eating Disorder Examination-Questionnaire: A Systematic Review of the Literature” [32] received the most citations. In the final period, studies involving systematic reviews and meta-analyses were often cited, as were those with broader research topics (e.g., ethnic groups and the Internet).

**Figure 3:**
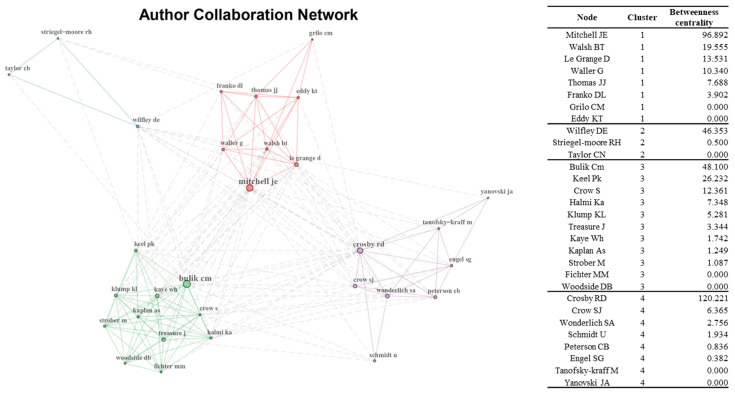
Author collaboration network.

Author collaboration was visualized using the top 30 authors with the highest betweenness centrality scores to display collaborative relationships among researchers (Figure 3). Betweenness centrality in the author collaboration network represents the researcher’s capacity to influence other researchers and spread information quickly [33]. The size and label of nodes are proportional to the frequency of each node in the author collaboration network. This means that authors with larger node sizes and labels often collaborate, and these researchers quickly transfer scientific knowledge. As a result, four clusters were found, centered on researchers with high betweenness centrality: Mitchell, JE in Cluster 1; Wifley, DE in Cluster 2; Builk, CM in Cluster 3; and Crosby RD in Cluster 4.

**Figure 4:**
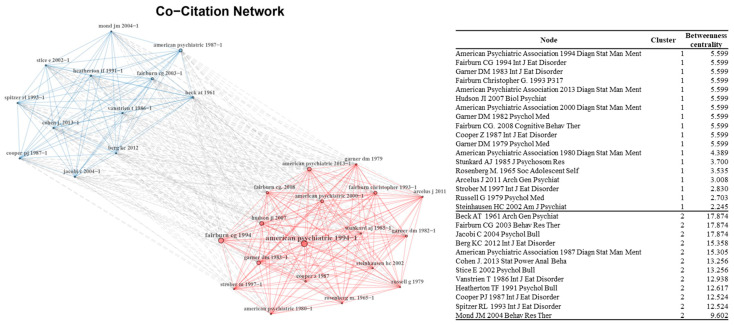
Co-citation network.

In addition, a co-citation network was drawn to identify the relationships among the representative sources frequently cited by articles of *Eating Disorders* (Figure 4). Each node of the co-citation network represents a cited reference source, and links between nodes are created if the corresponding nodes are cited by the same source. Articles frequently cited in the same journal tend to be densely networked. Densely connected nodes are grouped into the same cluster, and each cluster often shares similarities in terms of research topics. In this study, two large communities were discovered. The references in community one mainly focused on the assessment of eating disorders and clinical features (e.g., [30,34,35,36,37]). The references in community 2 are mainly about theory building and tool development (e.g., [32,38,39,40]).

### 3.3. Characteristics of Research Topics

#### 3.3.1. Discovery of Prominent Research Topics

Topic modeling and topic network analysis revealed the 47 most prominent research topics and their associations (Table 2) and the representative article for each topic (Table 3). At the topic level, technology (Topic 5 and T5) was the most popular among the 47 most salient research topics, accounting for approximately 3.8% of the total topic weight. The top words for that topic were “Internet”, “online”, “professional”, “technology”, and “international”. The article most closely associated with that topic was “User-Centered Design for Technology-Enabled Services for Eating Disorders [41]”. This result indicates online space became an important medium for ED diagnosis and clinical practices.

Dieting (T22) and BN (T18) were also widely studied topics, accounting for approximately 3.2% of the total topic weight. This result indicates that many researchers were interested in dieting (related to weight evaluation) as well as BN. An article related to dieting is “Eating Disorders, Dieting, and the Accuracy of Self-Reported Weight” [42]. The popularity of dieting topic demonstrated that many ED researchers found self-evaluation of body weight or excessive weight control relevant to EDs.

One article associated with BN is “Comparative Validity of the Chinese Versions of the Bulimic Inventory Test Edinburgh and Eating Attitudes Test for DSM-IV Eating Disorders Among High School Dance and Nondance Students in Taiwan” [43]. Compared to other ED topics (e.g., BED [T29], AN [T20], ARFID [T15]), BN was most widely studied in *IJED*. However, it should be noted that Topic 29 (BED) and Topic 1 (EDNOS) were related to binge eating, and these two topics accounted for about 6% of the overall topic weight, which is larger than BN. As shown in Table 2, both Topic 29 (BED) and Topic 1 (EDNOS) contained keywords related to binge eating. Topic 29 and Topic 1 may diverge due to revisions in the definition of BED in the *Diagnostic and Statistical Manual of Mental Disorders* (DSM-IV) and DSM-5. In the fourth edition of the DSM-IV, BED was classified as an Autonomous Eating Disorder not Otherwise Specified (EDNOS) [44]. In the fifth edition of the *Diagnostic and Statistical Manual of Mental Disorders* (DSM-5) published in May 2013, BED was listed in addition to other eating disorder diagnoses, BN and AN [45,46]. As this study targeted the ED research over three decades, those topics related to binge eating may have been classified into the eating behavior group rather than ED symptoms. In summary, the most researched ED-related topics in *IJED* were BED (accounting for nearly 6%), BN (3.2%), AN (2.8%), and ARFID (1.7%).

#### 3.3.2. Research Topic Network

**Figure 5:**
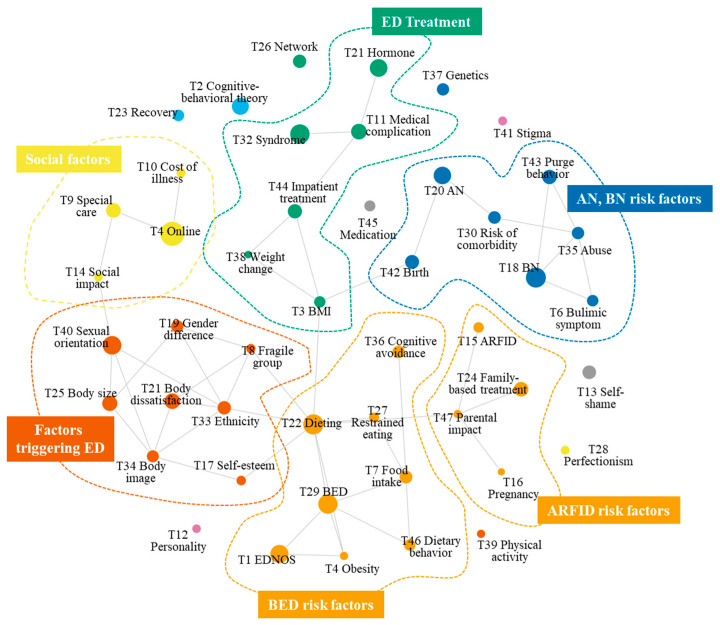
Topic network result.

Some topics tended to have overlapped themes and characteristics. Based on the degree of similarities shared by the topics, topic correlations were estimated and topic network structures were identified (Figure 5). As a result of the topic network analysis and community detection, six groups of 37 of the 47 topics were produced, leaving 10 stand-alone topics. The groups included BED risk factors (Group 1), factors triggering ED (Group 2), AN, BN risk factors (Group 3), treatment (Group 4), social factors (Group 5), and ARFID risk factors (Group 6). Groups 1, 3, and 6 were formed by connecting important risk factors with an emphasis on key EDs. Group 1 comprised, for example, BED-related topics and risk factors that are frequently studied in the context of BED.

The group with the most significant total topic weight, accounting for approximately 18.3% of the total topic weight, was mostly related to BED risk factors: EDNOS (T1), obesity (T4), food intake (T7), dieting (T22), restrained eating (T27), BED (T29), cognitive avoidance (T36), and eating behavior on mood (T46). The close link between obesity (T4) and two binge eating topics (T1 and T29) was found, which demonstrated that many ED researchers were interested in the effects of obesity on binge eating. For instance, Amianto, Ottone, Abbate Daga, and Fassino [44] conducted a systematic review study with binge eating research and many studies were conducted with obese population. Similarly, the edge of BED topic (T29) was connected to food intake (T7), dieting (T22), and dietary behavior (T46), showing that much research examined the effects of food behaviors on BED.

Another major group, accounting for approximately 17.4% of the total topic weight, was the factors triggering EDs. In that group, research topics included the effects of gender (T19 and T40), body image and self-esteem (T17, T25, and T34), internalization (T31), ethnicity (T33), and groups at risk of ED (T8). T19 and T40 dealt with gender issues, but their research foci differed. Studies related to T40 (labeled as gender/gender identity) examined whether biological gender or gender identity can influence EDs, whereas T19 (labeled as a gender stereotype) questioned the impact of social preconceptions about gender attributes, such as masculinity and femininity, on EDs.

Group 3 was labeled as “AN, BN risk factor”, accounting for 16.4% of the total topic weight. This group consists of bulimic symptoms (T6), BN (T18), AN (T20), risk of comorbidity (T30), abuse (T35), birth (T42), and purge behavior (T43). We found a close relationship between AN (T20) and the birth topic (T42), indicating that many researchers examined the effects of birth-related issues on AN. The close relationship between these two topics can be supported by many previous studies examining the relationships between birth patterns and AN [91,92]. Similarly, the abuse topic (T35) was closely related to bulimic symptoms (T6) and BN (T18) and purge behavior (T43). The results may indicate that researchers who investigated BN and purging disorder frequently considered various forms of abuses, such as sexual [93], physical [94], emotional [95], and substance abuse [96].

Other topic groups included ED treatment (13.7%), social factors (9.1%), and family-related factors (6.6%). ARFID was found to be often studied with the family-based treatment (FBT) topic (T24). Several previous studies suggested that FBT could be used to treat people with ARFID [97,98], which explains the close connection between the ARFID (T15) topic and FBT (T24). FBT is also linked to the parent effect topic (T47), indicating that ARFID was frequently considered in the context of the family.

#### 3.3.3. Classification of Research Topics by Overall Popularity and Historical Trend

**Figure 6:**
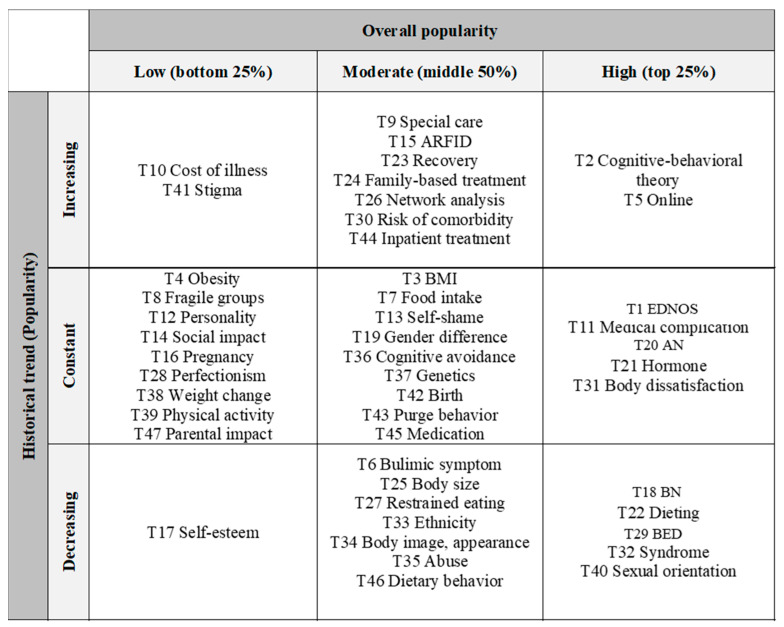
Classification of research topics by overall popularity and popularity trend.

Research topics were classified according to historical trends and overall popularity based on two metrics: changes in topic weights and expected topic estimates (Table 4). In addition, topics were grouped using a combination of overall popularity and historical trends in topic popularity (see Figure 6).

In terms of historical trends, the following 11 topics were classified into “increasing” as their topic weights have increased over time: cognitive-behavioral theory (T2), online (T4), special care (T9), cost of illness (T10), ARFID (T15), recovery (T23), family-based treatment (T24), network analysis (T26), risk of comorbidity (T30), stigma (T41), and inpatient treatment (T44). In particular, popularity of topics belonging to Group 5 (social factor) and Group 6 (family) tend to increase over time, considering the topic weights of three topics out of four topics in Group 5 (social factor) and two topics out of four topics in Group 6 (family) were classified into “increasing” in historical trends.

The topic weights of the following 13 topics tend to be “decreasing”: bulimic symptoms (T6), self-esteem (T17), BN (T18), dieting (T22), body size (T25), restrained eating (T27), overeating (T29), syndrome (T32), ethnicity (T33), body image, appearance (T34), abuse (T35), sexual orientation (T40), and dietary behavior (T46). This trend was evident in the topics of Group 2 (factors triggering ED), as the topic weights of six out of eight topics decreased.

Finally, 23 subjects were classified as “constant” in the historical trends because there was no significant difference in topic weights over the three decades. These topics included binge-eating diagnosis (T1), BMI (T3), obesity (T4), food intake (T7), fragile groups (T8), medical complications (T11), personality (T12), self-shame (T13), social impact (T14), pregnancy (T16), gender differences (T19), AN (T20), hormones (T21), perfectionism (T28), body dissatisfaction (T31), cognitive avoidance (T36), genetics (T37), weight change (T38), physical activity (T39), birth (T42), purge behavior (T43), medication (T45), and parental impact (T47).

Expected topic weights were considered to determine the overall popularity of the topic. The following 12 topics were in the top 25th percentile of the median topic weights: binge-eating diagnosis (T1), cognitive-behavioral theory (T2), online (T5), medical complications (T11), BN (T18), AN (T20), hormones (T21), dieting (T22), overeating (T29), body dissatisfaction (T31), syndrome (T32), and sexual orientation (T40). The results show that many ED studies on treatment have been conducted, given that three out of six topics in Group 4 (treatment) were classified as “high” in the overall popularity classification.

The following 12 topics were in the bottom 25th percentile of the median topic weights, meaning they have been understudied compared to other major topics: obesity (T4), fragile groups (T8), cost of illness (T10), personality (T12), social impact (T14), pregnancy (T16), self-esteem (T17), perfectionism (T28), weight change (T38), physical activity (T39), stigma (T41), and parental impact (T47).

## 4. Discussion

This study implemented bibliometric analysis and a text mining approach to answer three major research questions. To answer RQ1, this study identified the general characteristics of ED studies. We found that the number of articles published in *Eating Disorders* has grown steadily. This indicates that the importance of ED topics has escalated, and each paper published in *Eating Disorders* has received more attention from researchers than in the past.

The main goal of RQ2 is to identify how ED research was developed, and citation patterns were examined to answer three specific research questions. As the first step of citation analysis, this study pinpointed articles that received the most attention from fellow researchers interested in EDs in the first (1990–1999), second (2000–2009), and third decade (2010–2021) of the ED research and how those articles served as guidance on their own. Among the articles published in the first decade, articles concerning assessment tool development received many citations. In the second decade, systematic review and meta-analysis studies that summarize the past ED research outcomes and propose future research directions were cited frequently. In the third decade, the popularity of studies using systematic reviews and meta-analysis remained high, but internet-based studies also drew a lot of interest from academics. This finding implies that research that serves as the foundation for further investigations and summarizes previous research outcomes is widely cited. However, such citation patterns may change over time.

Secondly, the author collaboration network was examined. The author collaboration network allows tracing collaborative efforts devoted to ED research. This result could show how knowledge is disseminated among researchers in developing ED research and the researchers who played a critical role in spreading knowledge. Specifically, we discovered four major hubs of the ED research in the author collaboration network. The prolific authors were centered in the network.

The final step of citation analysis was co-citation network analysis. The co-citation network reveals the key articles or documents that establish the foundation of ED research. In addition to academic research published in academic journals, many studies frequently cited all editions of handbooks of “*Diagnostic and Statistical Manual of Mental Disorders*” by the American Psychiatric Association. This handbook is commonly used in the United States for psychiatric illness diagnosis. High centrality scores of these handbooks indicate that ED diagnosis is an important part of the ED research. By examining the associations among these cited references, this study also discovered salient research themes that underpin ED research. One stream of research themes was related to ED-related theories and tool development, and the other was related to the diagnosis and treatment of ED. This implies that articles on eating disorders are concerned with both the theoretical and clinical features.

To answer RQ3 regarding the research topic landscape, this study applied topic modeling and topic network analysis. We discovered the 47 most outstanding topics and the associations among these topics by examining the similarities among the ED research topics. As a result of the topic clustering, we found that ED researchers were particularly interested in the relationships between key EDs and risk factors. Based on the keyword network analysis, Shah, Ahmad, Khan, and Sun [19] discovered that BN and AN frequently appeared in the top 100 ED articles that are frequently cited. Alongside this previous finding, this study discovered that ED topics played an important role in the research topic clusters by linking ED-related risk factors. As a result of topic clustering, we found that EDs were studied in different contexts and variables. Many BED studies, for example, focused on eating behaviors and dietary patterns, while the effects of family-related factors on ARFID were often examined. Moreover, many AN studies focused on birth-related issues and various types of abuse were examined to comprehend BN.

Beyond that, our study revealed both snapshots and the evolution of research topics related to EDs frequently studied by researchers. This study utilized two indicators, overall topic popularity and historical trends of topic popularity, to demonstrate the progress of research development for specific research topics and track the varying popularity of each research topic over time. Higher societal and academic demands on a particular subject may lead researchers to investigate the related topic more actively than in the past. A recent bibliometric study on ED research [99] revealed that ED researchers’ interest in ED treatment has been steadily increasing. Compared to previous findings, our study can demonstrate more specific results. For example, we discovered that cognitive-behavioral theory is popular among ED researchers and its popularity is growing. In addition, we found that the overall popularity of AN was high and the popularity of this topic tends to be constant. The overall popularity of BN and BED were high, but weights of these topics tend to decrease over time. On the contrary, the overall popularity of ARFID was moderate but the popularity tends to increase over time. This result indicates that AN, BN, and BED were extensively investigated. However, BN and BED were less studied than the past as interest in ARFID grows. According to previous research in India [16], AN was the most extensively studied in India ED literature, followed by BED and BN. Similar to our findings, the share of BN research decreased over time, while the popularity of AN and BED increased significantly [16]. Based on our findings, young researchers may need to pay closer attention to these research topics, which have received more attention from ED researchers than in the past. In contrast, some topics were understudied and thus had much room for contribution, which requires more attention from researchers for the sustainable and continuous development of ED research.

## 5. Conclusions

This study aimed to illustrate the evolution of the articles of *Eating Disorders*, a leading peer-reviewed, SSCI-indexed journal for nutrition and dietetics, psychiatry, and psychology since 1990, by applying a computer-assisted bibliometric approach combined with text mining. In the process, we analyzed the major attributes of the journal, including authors, citations, and characteristics of research topics, and compared the results over three decades.

Our summary of key articles and authors in the field may facilitate a search for fundamental concepts or results prevalent in the previous ED research. Our findings regarding the research topic network demonstrated the topics that researchers and clinicians frequently considered together. For instance, a particular risk factor, such as abuse, was often studied together with BN. Based on this result, researchers and clinicians may connect the dots with regard to the evaluation of particular risk factors in different types of EDs that are understudied. Our findings concerning changes in topics published across three decades of the articles demonstrated that the popularity of research topics has evolved over time. Often the researchers choose research topics from the socially sensitive and pressing issues. Given that research topics that are actively studied can demonstrate the socially relevant ED issues within each time period, our results can benefit researchers to comprehend specific ED issues that are considered important. Clinicians and researchers can also use the summary to identify important topics related to EDs that have been continually studied by researchers or important but understudied topics for further development in the field.

Despite contributions, our study had several limitations and thus we encourage future research directions to overcome the limitations of this study. Firstly, this study chose only one journal for analysis. However, as mentioned in the methodology, there are many prestigious ED-related journals and other journals that publish ED studies. Hence, our findings may not be representative. Still, our findings can be important empirical evidence to understand ED research trends over time. Secondly, this study utilized the machine learning algorithm to identify salient ED-related research topics and to detect the relationships among them. This approach can demonstrate which topics were frequently studied together in the empirical research. However, this approach may not be consistent with the existing studies grounded on the formal classification system and frameworks. Hence, future studies need to compare the results derived from the machine learning approach and expert classifications. Thirdly, since bibliometrics are highly influenced by the quality of the database, our results could have been similarly influenced as well. For instance, WoS does not provide links or information to track authors who may have changed their names. This study focused on author collaboration networks rather than examining the general statistics of the authors to overcome this problem. Future studies need to examine the impact of authors in the ED research. Finally, we analyzed the articles according to their titles, keywords, and abstracts using an automated text mining approach. Although, such data points contain essential information about the articles and offer a good summary, more specific information (e.g., methodology used and participant profile) was inaccessible and should be considered in future analyses of the development of studies on EDs.

## Figures and Tables

**Table 1:** Top 20 most cited papers published in each decade.

Period	First Author	Year	Title	Citation Counts
First decade: 1990–1999	Fairburn, C.G.	1994	Assessment of Eating Disorders—Interview or Self-Report Questionnaire	2998
	Luce, K.H.	1999	The Reliability of the Eating Disorder Examination-Self-Report Questionnaire Version (EDE-Q)	628
	Spitzer, R.L.	1993	Binge Eating Disorder—Its Further Validation in a Multisite Study	586
	Strober, M.	1997	The Long-Term Course of Severe Anorexia Nervosa in Adolescents: Survival Analysis of Recovery, Relapse, and Outcome Predictors Over 10–15 Years in a Prospective Study	586
	Spitzer, R.L.	1992	Binge Eating Disorder—a Multisite Field Trial of the Diagnostic-Criteria	561
	Cash, T.F.	1997	The Nature and Extent of Body-Image Disturbances in Anorexia Nervosa and Bulimia Nervosa: A Meta-Analysis	460
	Collins, M.E.	1991	Body Figure Perceptions and Preferences Among Preadolescent Children	437
	Arnow, B.	1995	The Emotional Eating Scale—the Development of a Measure to Assess Coping with Negative Affect by Eating	426
	Heinberg, L.J.	1995	Development and Validation of the Sociocultural Attitudes Towards Appearance Questionnaire	397
	Wiseman, C.V.	1992	Cultural Expectations of Thinness in Women—An Update	382
	Killen, J.D.	1994	Pursuit of Thinness and Onset of Eating Disorder Symptoms in a Community Sample of Adolescent Girls—a 3-Year Prospective Analysis	361
	Holderness, H.C.	1994	Co-Morbidity of Eating Disorders and Substance-Abuse Review of the Literature	309
	Bryantwaugh, R.J.	1996	The Use of the Eating Disorder Examination with Children: A Pilot Study	300
	Pope, H.G.	1999	Evolving Ideals of Male Body Image as Seen Through Action Toys	272
	Westenhoefer, J.	1999	Validation of the Flexible and Rigid Control Dimensions of Dietary Restraint	270
	Thompson, J.K.	1995	Development of Body-Image, Eating Disturbance, and General Psychological Functioning in Female Adolescents—Covariance Structure Modeling and Longitudinal Investigations	227
	Rosen, J.C.	1996	Body Shape Questionnaire: Studies of Validity and Reliability	220
	Serpell, L.	1999	Anorexia Nervosa: Friend or Foe?	220
	Thompson, J.K.	1991	Psychometric Qualities of the Figure Rating-Scale	219
Second decade: 2000–2009	Rucker, C.E.	1992	Body Images, Body-Size Perceptions, and Eating Behaviors among African-American and White College Women	215
	Groesz, L.M.	2002	The Effect of Experimental Presentation of Thin Media Images on Body Satisfaction: A Meta-Analytic Review	927
	Hoek, H.W.	2003	Review of the Prevalence and Incidence of Eating Disorders	821
	Thompson, J.K.	2004	The Sociocultural Attitudes Towards Appearance Scale-3 (SATAQ-3): Development and Validation	617
	Berkman, N.D.	2007	Outcomes of Eating Disorders: A Systematic Review of the Literature	328
	Smolak, L.	2000	Female Athletes and Eating Problems: A Meta-Analysis	320
	Bulik, C.M.	2007	Anorexia Nervosa Treatment: A Systematic Review of Randomized Controlled Trials	306
	Striegel-Moore, R.H.	2009	Gender Difference in the Prevalence of Eating Disorder Symptoms	287
	Presnell, K.	2004	Risk Factors for Body Dissatisfaction in Adolescent Boys and Girls: A Prospective Study	286
	Cash, T.F.	2004	The Assessment of Body Image Investment: An Extensive Revision of the Appearance Schemas Inventory	263
	Leit, R.A.	2001	Cultural Expectations of Muscularity in Men: The Evolution of Playgirl Centerfolds	262
	Cohane, G.H.	2001	Body Image in Boys: A Review of the Literature	259
	Peterson, C.B.	2007	Psychometric Properties of the Eating Disorder Examination-Questionnaire: Factor Structure and Internal Consistency	243
	Luce, K.H.	2008	Eating Disorder Examination Questionnaire (EDE-Q): Norms for Undergraduate Women	239
	O’Dea, J.A.	2000	Improving the Body Image, Eating Attitudes, and Behaviors of Young Male and Female Adolescents: A New Educational Approach That Focuses on Self-Esteem	235
	Fichter, M.M.	2006	Twelve-Year Course and Outcome Predictors of Anorexia Nervosa	228
	Katzman, D.K.	2005	Medical Complications in Adolescents with Anorexia Nervosa: A Review of the Literature	215
	Cash, T.F.	2002	The Impact of Body Image Experiences: Development of the Body Image Quality of Life Inventory	212
	Stice, E.	2000	Dissonance Prevention Program Decreases Thin-Ideal Internalization, Body Dissatisfaction, Dieting, Negative Affect, and Bulimic Symptoms: A Preliminary Experiment	211
	Bydlowski, S.	2005	Emotion-Processing Deficits in Eating Disorders	211
	Godart, N.T.	2002	Comorbidity Between Eating Disorders and Anxiety Disorders: A Review	208
Third decade: 2010–2021	Berg, K.C.	2012	Psychometric Evaluation of the Eating Disorder Examination and Eating Disorder Examination-Questionnaire: A Systematic Review of the Literature	494
	Gearhardt, A.N.	2012	An Examination of the Food Addiction Construct in Obese Patients with Binge Eating Disorder	269
	Keel, P.K.	2010	Update On Course and Outcome in Eating Disorders	240
	Marques, L.	2011	Comparative Prevalence, Correlates of Impairment, and Service Utilization for Eating Disorders Across US Ethnic Groups: Implications for Reducing Ethnic Disparities in Health Care Access for Eating Disorders	222
	Vall, E.	2015	Predictors of Treatment Outcome in Individuals with Eating Disorders: A Systematic Review and Meta-Analysis	214
	Tiggemann, M.	2013	Netgirls: The Internet, Facebook, and Body Image Concern in Adolescent Girls	211
	Allison, K.C.	2010	Proposed Diagnostic Criteria for Night Eating Syndrome	194
	Smink, F.R.E.	2014	Prevalence and Severity of DSM-5 Eating Disorders in a Community Cohort of Adolescents	193
	Couturier, J.	2013	Efficacy of Family-Based Treatment for Adolescents with Eating Disorders: A Systematic Review and Meta-Analysis	172
	Vocks, S.	2010	Meta-Analysis of the Effectiveness of Psychological and Pharmacological Treatments for Binge Eating Disorder	171
	Hay, P.	2013	A Systematic Review of Evidence for Psychological Treatments in Eating Disorders: 2005–2012	158
	Fichter, M.M.	2016	Mortality in Eating Disorders—Results of a Large Prospective Clinical Longitudinal Study	156
	Bryant-Waugh, R.	2010	Feeding and Eating Disorders in Childhood	155
	Wildes, J.E.	2010	Emotion Avoidance in Patients with Anorexia Nervosa: Initial Test of a Functional Model	145
	Rohde, P.	2015	Development and Predictive Effects of Eating Disorder Risk Factors during Adolescence: Implications for Prevention Efforts	137
	Mabe, A.G.	2014	Do You “Like” My Photo? Facebook Use Maintains Eating Disorder Risk	136
	Keel, P.K.	2013	Psychosocial Risk Factors for Eating Disorders	131
	Tasca, G.A.	2014	Attachment and Eating Disorders: A Review of Current Research	112
	Mclean, S.A.	2015	Photoshopping the Selfie: Self Photo Editing and Photo Investment Are Associated with Body Dissatisfaction in Adolescent Girls	112
	Hill, L.S.	2010	SCOFF, the Development of An Eating Disorder Screening Questionnaire	111

**Table 2:** Topic summary.

Group Label	Topic Name	Top Words	Topic Weight
BED risk factors	T22 Dieting	weight, diet, overweight	0.032
	T29 BED	binge eating, obese, binge eater	0.031
	T1 EDNOS	bed, ednos, subthreshold	0.029
	T7 Food intake	food, activation, craving	0.021
	T36 Cognitive avoidance	cognitive, avoidance, deficit	0.020
	T46 Eating behavior on mood	mood, intake, fat	0.019
	T27 Restrained eating	restraint, shape, stroop	0.017
	T4 Obesity	obesity, time, sleep	0.014
Factors triggering ED	T40 Sexual orientation	woman, heterosexual, man	0.030
	T31 Internalization	peer, internalization, body_dissatisfaction	0.025
	T25 Body size	body, size, image	0.025
	T33 Ethnicity	Asian, American, ethnic	0.022
	T19 Gender	female, male, gender	0.020
	T34 Body image, appearance	body_image, appearance, disturbance	0.020
	T17 Self-esteem	esteem, self, low_self	0.016
	T8 Fragile groups	athlete, student, school	0.016
AN, BN risk factors	T18 BN	bulimia, nervosa, dsm	0.032
	T20 AN	anorexia nervosa, death, psychiatric	0.028
	T43 Purge behavior	purge, frequency, purging	0.023
	T42 Birth	bone, bear, birth	0.022
	T30 Risk of comorbidity	risk, comorbid, suicide	0.020
	T35 Abuse	abuse, impulsivity, sexual_abuse	0.020
	T6 Bulimic symptom	bulimic, bulimic_symptom, anger	0.019
Treatment	T32 Syndrome	syndrome, anorexic, deficiency	0.031
	T21 Metabolism	serum, brain, concentration	0.028
	T11 Medical complication	medical, complication, anorexia nervosa	0.025
	T44 Inpatient treatment	admission, discharge, inpatient	0.023
	T3 BMI	bmi, body_mass, index_bmi	0.018
	T38 Weight change	weight, relapse, gain	0.012
Social factors	T5 Technology	internet, online, professional	0.038
	T9 Special care	interpersonal, carer, skill	0.024
	T10 Economic impact	cost, healthcare, economic	0.015
	T14 Social impact	social, distress, sexual	0.014
ARFID risk factors	T24 Family-based treatment	adolescent, family, fbt	0.023
	T15 ARFID	arfid, avoidant, sensitivity	0.017
	T47 Parental impact	parent, parental, child	0.014
	T16 Pregnancy	pregnancy, mother, maternal	0.012
Stand-alone	T23 Recovery	recovery, motivation, recover	0.019
	T26 Network analysis	network, fear, strength	0.022
	T28 Perfectionism	perfectionism, obsessive, compulsive	0.015
	T37 Genetics	twin, genetic, lifetime	0.020
	T39 Physical activity	exercise, physical_activity, physical	0.014
	T41 Stigma	stigma, youth, barrier	0.015
	T45 Medication	medication, placebo, open	0.018
	T2 Cognitive-behavioral	cbt, cognitive, clinician	0.027
	T12 Personality	personality, anxiety, attachment	0.014
	T13 Self-shame	self, shame, feeling	0.022

**Table 3:** Representative articles highly associated with each topic.

Group Label	Topic Name	Representative Article: Title (References)
BED risk factors	T22 Dieting	Eating disorders, dieting, and the accuracy of self-reported weight [42]
	T29 BED	Hunger and binge eating: a meta-analysis of studies using ecological momentary assessment [47]
	T1 EDNOS	Comparison of DSM-IV versus proposed DSM-5 diagnostic criteria for eating disorders: Reduction of eating disorder not otherwise specified and validity [48]
	T7 Food intake	Forbidden fruit: Does thinking about a prohibited food lead to its consumption? [49]
	T36 Cognitive avoidance	Cognitive avoidance of threat cues: Association with eating disorder inventory scores among a non-eating-disordered population [50]
	T46 Eating behavior on mood	Effects of eating behavior on mood: a review of the literature [51]
	T27 Restrained eating	Dietary restraint and addictive behaviors: The generalizability of Tiffany’s cue reactivity model [52]
	T4 Obesity	Time trends in obesity and eating disorders [53]
Factors triggering ED	T40 Sexual orientation	A comparison of lesbians, gay men, and heterosexuals on weight and restrained eating [54]
	T31 Internalization	Do universal media literacy programs have an effect on weight and shape concern by influencing media internalization? [55]
	T25 Body size	Experimental manipulation of visual attention affects body size adaptation but not body dissatisfaction [56]
	T33 Ethnicity	The impact of racial stereotypes on eating disorder recognition [57]
	T19 Gender	The development and validation of the muscularity-oriented eating test: A novel measure of muscularity-oriented disordered eating [58]
	T34 Body image, appearance	Body image, social comparison, and eating disturbance: A covariance structure modeling investigation [59]
	T17 Self-esteem	Kindness begins with yourself: The role of self-compassion in adolescent body satisfaction and eating pathology [60]
	T8 Fragile groups	Female athletes and eating problems: A meta-analysis [61]
AN, BN risk factors	T18 BN	Comparative validity of the Chinese versions of the bulimic inventory test Edinburgh and eating attitudes test for DSM-IV eating disorders among high school dance and nondance students in Taiwan [43]
	T20 AN	An audit of a British sample of death certificates in which anorexia nervosa is listed as a cause of death [62]
	T43 Purge behavior	The use of multiple purging methods as an indicator of eating disorder severity [63]
	T42 Birth	Season of birth bias and anorexia nervosa: Results from an international collaboration [64]
	T30 Risk of comorbidity	Suicidality in adolescents and adults with binge-eating disorder: Results from the national comorbidity survey replication and adolescent supplement [65]
	T35 Abuse	Trait-defined eating disorder subtypes and history of childhood abuse [66]
	T6 Bulimic symptom	Anger and bulimic psychopathology among nonclinical women [67]
Treatment	T32 Syndrome	A case report of Usher’s syndrome and anorexia nervosa [68]
	T21 Metabolism	Plasma tryptophan levels and anorexia in liver cirrhosis [69]
	T11 Medical complication	Digestive complication in severe malnourished anorexia nervosa patient: a case report of necrotizing colitis [70]
	T44 Inpatient treatment	A naturalistic comparison of two inpatient treatment protocols for adults with anorexia nervosa: Does reducing duration of treatment and external controls compromise outcome? [71]
	T3 BMI	Body composition and menstrual status in adults with a history of anorexia nervosa-at what fat percentage is the menstrual cycle restored? [72]
	T38 Weight change	Elevated pre-morbid weights in bulimic individuals are usually surpassed post-morbidly: Implications for perpetuation of the disorder [73]
Social factors	T5 Technology	User-centered design for technology-enabled services for eating disorders [41]
	T9 Special care	The use of guidelines for dissemination of “best practice” in primary care of patients with eating disorders [74]
	T10 Economic impact	Key factors that influence government policies and decision making about healthcare priorities: Lessons for the field of eating disorders [75]
	T14 Social impact	Eating disorders treatment experiences and social support: Perspectives from service seekers in mainland China [76]
ARFID risk factors	T24 Family-based treatment	Family-based treatment: Where are we and where should we be going to improve recovery in child and adolescent eating disorders [77]
	T15 ARFID	Development of the Pica, ARFID, and Rumination Disorder Interview, a multi-informant, semi-structured interview of feeding disorders across the lifespan: A pilot study for ages 10–22 [78]
	T47 Parental impact	The relationship between parent feeding styles and general parenting with loss of control eating in treatment-seeking overweight and obese children [79]
	T16 Pregnancy	Documenting the course of loss of control over eating prior to, during and after pregnancy among women with pre-pregnancy overweight and obesity [80]
Stand-alone	T23 Recovery	# recovery: Understanding recovery from the lens of recovery-focused blogs posted by individuals with lived experience [81]
	T26 Network analysis	Network analysis: An innovative framework for understanding eating disorder psychopathology [82]
	T28 Perfectionism	Stress situation reveals an association between perfectionism and drive for thinness [83]
	T37 Genetics	Twin studies and the etiology of eating disorders [84]
	T39 Physical activity	Monitoring eating and activity: Links with disordered eating, compulsive exercise, and general wellbeing among young adults [85]
	T41 Stigma	Interventions to reduce the stigma of eating disorders: A systematic review and meta-analysis [86]
	T45 Medication	Bulimia nervosa treatment: A systematic review of randomized controlled trials [87]
	T2 Cognitive-behavioral	Patients’ experiences of brief cognitive-behavioral therapy for eating disorders: A qualitative investigation [88]
	T12 Personality	Relationships among attachment styles, personality characteristics, and disordered eating [89]
	T13 Self-shame	Within-persons predictors of change during eating disorders treatment: An examination of self-compassion, self-criticism, shame, and eating disorder symptoms [90]

**Table 4:** Expected topic weight comparisons over three decades.

		1990–1999	2000–2009	2010–2021	Difference in Topic Estimate	A Two-Sided 95% Confidence Interval							Historical	Overall
						90s–00s		90s–10s		00s–10s				
	Topic Label	Topic Estimate				ci. Lower	ci. Upper	ci. Lower	ci. Upper	ci. Lower	ci. Upper	Topic Weight	Trend Classification	Popularity Classification
BED risk factors	T1 EDNOS	0.024	0.026	0.028	0.002	0.007	−0.002	0.007	−0.002	0.007	−0.002	0.029	Constant	High
	T4 Obesity	0.020	0.018	0.016	−0.002	0.002	−0.005	0.002	−0.005	0.002	−0.005	0.014	Constant	Low
	T7 Food intake	0.019	0.020	0.020	0.001	0.004	−0.003	0.005	−0.003	0.004	−0.003	0.021	Constant	Moderate
	T22 Dieting	0.031	0.025	0.019	−0.006	−0.003	−0.009	−0.003	−0.010	−0.003	−0.009	0.032	Decreasing	High
	T27 Restrained eating	0.028	0.020	0.011	−0.009	−0.005	−0.012	−0.005	−0.012	−0.005	−0.012	0.017	Decreasing	Moderate
	T29 BED	0.034	0.028	0.021	−0.006	−0.002	−0.011	−0.003	−0.011	−0.003	−0.011	0.031	Decreasing	High
	T36 Cognitive avoidance	0.019	0.021	0.023	0.002	0.006	−0.002	0.006	−0.002	0.006	−0.002	0.020	Constant	Moderate
	T46 Dietary behavior	0.022	0.018	0.014	−0.004	0.000	−0.007	−0.001	−0.008	−0.001	−0.007	0.019	Decreasing	Moderate
Factors triggering ED	T8 Fragile groups	0.017	0.017	0.017	0.000	0.004	−0.003	0.004	−0.003	0.004	−0.003	0.016	Constant	Low
	T17 Self-esteem	0.024	0.019	0.013	−0.005	−0.002	−0.009	−0.002	−0.009	−0.002	−0.009	0.016	Decreasing	Low
	T19 Gender difference	0.021	0.020	0.019	−0.001	0.003	−0.004	0.003	−0.004	0.003	−0.004	0.020	Constant	Moderate
	T25 Body size	0.028	0.023	0.017	−0.006	−0.002	−0.010	−0.002	−0.010	−0.002	−0.010	0.025	Decreasing	Moderate
	T31 Body dissatisfaction	0.021	0.024	0.027	0.003	0.008	−0.001	0.007	−0.001	0.007	−0.002	0.025	Constant	High
	T33 Ethnicity	0.026	0.022	0.018	−0.004	0.000	−0.008	0.000	−0.008	0.000	−0.009	0.022	Decreasing	Moderate
	T34 Body image	0.026	0.020	0.015	−0.006	−0.002	−0.009	−0.002	−0.009	−0.002	−0.009	0.020	Decreasing	Moderate
	T40 Sexual orientation	0.029	0.024	0.020	−0.004	−0.001	−0.007	−0.001	−0.007	−0.001	−0.008	0.030	Decreasing	High
AN, BN risk factors	T6 Bulimic symptoms	0.033	0.022	0.011	−0.011	−0.007	−0.015	−0.007	−0.015	−0.007	−0.015	0.019	Decreasing	Moderate
	T18 BN	0.043	0.029	0.014	−0.015	−0.011	−0.018	−0.011	−0.018	−0.011	−0.018	0.032	Decreasing	High
	T20 AN	0.033	0.033	0.032	−0.001	0.004	−0.007	0.004	−0.007	0.004	−0.007	0.028	Constant	High
	T30 Risk of comorbidity	0.014	0.020	0.025	0.006	0.010	0.002	0.010	0.002	0.009	0.002	0.020	Increasing	Moderate
	T35 Abuse	0.028	0.022	0.015	−0.006	−0.003	−0.010	−0.003	−0.010	−0.003	−0.010	0.020	Decreasing	Moderate
	T42 Birth	0.022	0.024	0.026	0.002	0.006	−0.002	0.006	−0.002	0.006	−0.002	0.022	Constant	Moderate
	T43 Purge behavior	0.018	0.021	0.025	0.004	0.007	0.000	0.007	0.000	0.007	0.000	0.023	Constant	Moderate
Treatment	T3 BMI	0.015	0.018	0.021	0.003	0.006	0.000	0.006	0.000	0.006	0.000	0.018	Constant	Moderate
	T11 Complication	0.026	0.026	0.026	0.000	0.004	−0.005	0.004	−0.004	0.004	−0.004	0.025	Constant	High
	T21 Hormone	0.031	0.029	0.027	−0.002	0.003	−0.008	0.003	−0.007	0.003	−0.008	0.028	Constant	High
	T32 Syndrome	0.043	0.031	0.019	−0.012	−0.007	−0.017	−0.007	−0.017	−0.007	−0.017	0.031	Decreasing	High
	T38 Weight change	0.018	0.016	0.014	−0.002	0.001	−0.005	0.001	−0.005	0.001	−0.005	0.012	Constant	Low
	T44 Inpatient treatment	0.013	0.021	0.028	0.007	0.011	0.003	0.012	0.003	0.011	0.003	0.023	Increasing	Moderate
Social factors	T5 Online	0.020	0.033	0.045	0.013	0.019	0.007	0.019	0.007	0.019	0.007	0.038	Increasing	High
	T9 Special care	0.017	0.022	0.028	0.006	0.010	0.001	0.010	0.001	0.010	0.001	0.024	Increasing	Moderate
	T10 Cost of illness	0.010	0.017	0.025	0.007	0.012	0.004	0.011	0.004	0.011	0.004	0.015	Increasing	Low
	T14 Social impact	0.014	0.015	0.016	0.001	0.004	−0.002	0.004	−0.002	0.004	−0.002	0.014	Constant	Low
ARFID risk factors	T15 ARFID	0.007	0.017	0.027	0.010	0.014	0.006	0.014	0.006	0.014	0.006	0.017	Increasing	Moderate
	T16 Pregnancy	0.014	0.014	0.014	0.000	0.004	−0.003	0.004	−0.003	0.004	−0.003	0.012	Constant	Low
	T24 Family-based treatment	0.012	0.019	0.026	0.007	0.010	0.003	0.010	0.003	0.010	0.003	0.023	Increasing	Moderate
	T47 Parental impact	0.008	0.010	0.012	0.002	0.004	0.000	0.004	0.000	0.004	0.000	0.014	Constant	Low
Stand-alone	T2 Cognitive-behavioral theory	0.018	0.024	0.030	0.006	0.011	0.001	0.011	0.001	0.011	0.001	0.027	Increasing	High
	T12 Personality	0.018	0.019	0.019	0.000	0.004	−0.004	0.004	−0.004	0.004	−0.004	0.014	Constant	Low
	T13 Self-shame	0.021	0.022	0.022	0.000	0.004	−0.004	0.004	−0.004	0.004	−0.004	0.022	Constant	Moderate
	T23 Recovery	0.013	0.018	0.024	0.006	0.009	0.002	0.009	0.002	0.009	0.002	0.019	Increasing	Moderate
	T26 Network analysis	0.015	0.023	0.031	0.008	0.012	0.004	0.012	0.004	0.012	0.004	0.022	Increasing	Moderate
	T28 Perfectionism	0.019	0.018	0.017	−0.001	0.003	−0.005	0.003	−0.005	0.003	−0.005	0.015	Constant	Low
	T37 Genetics	0.023	0.021	0.020	−0.001	0.003	−0.006	0.003	−0.006	0.003	−0.006	0.020	Constant	Moderate
	T39 Physical activity	0.012	0.015	0.018	0.003	0.007	0.000	0.007	0.000	0.007	0.000	0.014	Constant	Low
	T41 Stigma	0.012	0.017	0.023	0.006	0.010	0.002	0.010	0.002	0.010	0.002	0.015	Increasing	Low
	T45 Medication	0.021	0.021	0.022	0.000	0.005	−0.004	0.005	−0.004	0.005	−0.004	0.018	Constant	Moderate

## Data Availability

Not applicable.
